# Maternal intake of dietary virgin coconut oil modifies essential fatty acids and causes low body weight and spiky fur in mice

**DOI:** 10.1186/s12906-017-1600-z

**Published:** 2017-01-28

**Authors:** Renuka Gunasekaran, Mohammed Rafid Shaker, Siti Waheeda Mohd-Zin, Aminah Abdullah, Azlina Ahmad-Annuar, Noraishah Mydin Abdul-Aziz

**Affiliations:** 1 Department of Parasitology, Faculty of Medicine, University of Malaya, 50603 Kuala Lumpur, Malaysia; 2 Department of Anatomy, Brain Korea 21 Program, Korea University College of Medicine, Seoul, 136-705 Korea; 3 School of Chemical Sciences and Food Technology, Faculty of Science and Technology, Universiti Kebangsaan Malaysia, Bangi, 43600 Selangor Malaysia; 4 Department of Biomedical Science, Faculty of Medicine, University of Malaya, 50603 Kuala Lumpur, Malaysia

**Keywords:** Coconut oil, Essential fatty acids, GCFID, Body weight, Spiky fur

## Abstract

**Background:**

Coconut oil is commonly used as herbal medicine worldwide. There is limited information regarding its effects on the developing embryo and infant growth.

**Methods:**

We investigated the effect of virgin coconut oil post-natally and until 6 weeks old in mice (age of maturity). Females were fed with either standard, virgin olive oil or virgin coconut oil diets 1 month prior to copulation, during gestation and continued until weaning of pups. Subsequently, groups of pups borne of the respective diets were continuously fed the same diet as its mother from weaning until 6 weeks old. Profiles of the standard and coconut oil diets were analysed by gas chromatography flame ionization detector (GCFID).

**Results:**

Analysis of the mean of the total weight gained/ loss over 6 weeks revealed that in the first 3 weeks, pups whose mothers were fed virgin coconut oil and virgin olive oil have a significantly lower body weight than that of standard diet pups. At 6 weeks of age, only virgin coconut oil fed pups exhibited significantly lower body weight. We report that virgin coconut oil modifies the fatty acid profiles of the standard diet by inducing high levels of medium chain fatty acids with low levels of essential fatty acids. Furthermore, pups borne by females fed with virgin coconut oil developed spiky fur.

**Conclusion:**

Our study has demonstrated that virgin coconut oil could affect infant growth and appearance via maternal intake; we suggest the use of virgin coconut oil as herbal medicine to be treated with caution.

## Background

Plants are considered the most common and valuable source of herbal medicine [1] and numerous therapeutic benefits from plants have been derived over centuries [2–4]. The use of herbal medicine during pregnancy revealed that between 7 and 55% of pregnant females take some form of herbal medicine [2, 4, 5]. Recent years have shown that the usage of herbal medicine in child-bearing women had become widespread for symptoms such as ease of delivery and pain management as it is assumed that it is safe [4, 6, 7]. There is however, a variation between cultures and ethnicities in the usage of herbal medicine [2, 8]. Women of child-bearing age recognize that chemicals in drugs as a potential danger but fail to appreciate the inherent dangers of herbal chemicals [4, 9]. However, several well-documented studies have found the association of herbal medicine as the cause of congenital malformations during pregnancy including embryo-toxicity [7, 10–12]. Both long term and short term use of herbal medicines before, during and after pregnancy causes low birth weight [13, 14], premature delivery [15], intrauterine growth retardation [16], decreased foetal survival rate [16] and increased foetal distress [17]. The effects of natural health products on foetal development, however, are poorly understood. The use of virgin olive oil as our control diet was on the basis of well-documented literature [18, 19] and it is also known to confer various health benefits including acting as an analgesic [20].

Countries within the Southeast Asian region are rich in coconut oil and other coconut by-products [21–23]. The coconut oil is extracted oil from the meat of the matured coconut [24]. Coconut oil is widely used during pregnancy in these countries where published studies on the use of herbal medicine during pregnancy has shown that in certain areas about 61% to 63.9% of women of child-bearing age used coconut oil as herbal medicine [1, 21, 25]. Coconut oil has been reported to facilitate delivery and prevent congenital malformations and/ or birth defects [1, 24].

Despite wanton usage of coconut oil, to our knowledge, there is no clear study which indicates and explains the benefits or danger of coconut oil treatment during pregnancy. Information concerning the potential effect of coconut oil on the growth and development of infants are lacking and has yet to be established. Thus, the objective of this study is to use an animal model to determine the effects of virgin coconut oil intake after pregnancy, and during the early weeks of the pups’ growth and development until age of maturity (6 weeks).

We asked whether dietary intake of virgin coconut oil fed to female mice prior to copulation, during copulation and during pregnancy could affect the weight of its progeny, and whether it could subsequently develop adverse effects in the later-life of offsprings with continued feeding of the virgin coconut oil diet.

## Methods

### Animal study

**Fig. 1:**
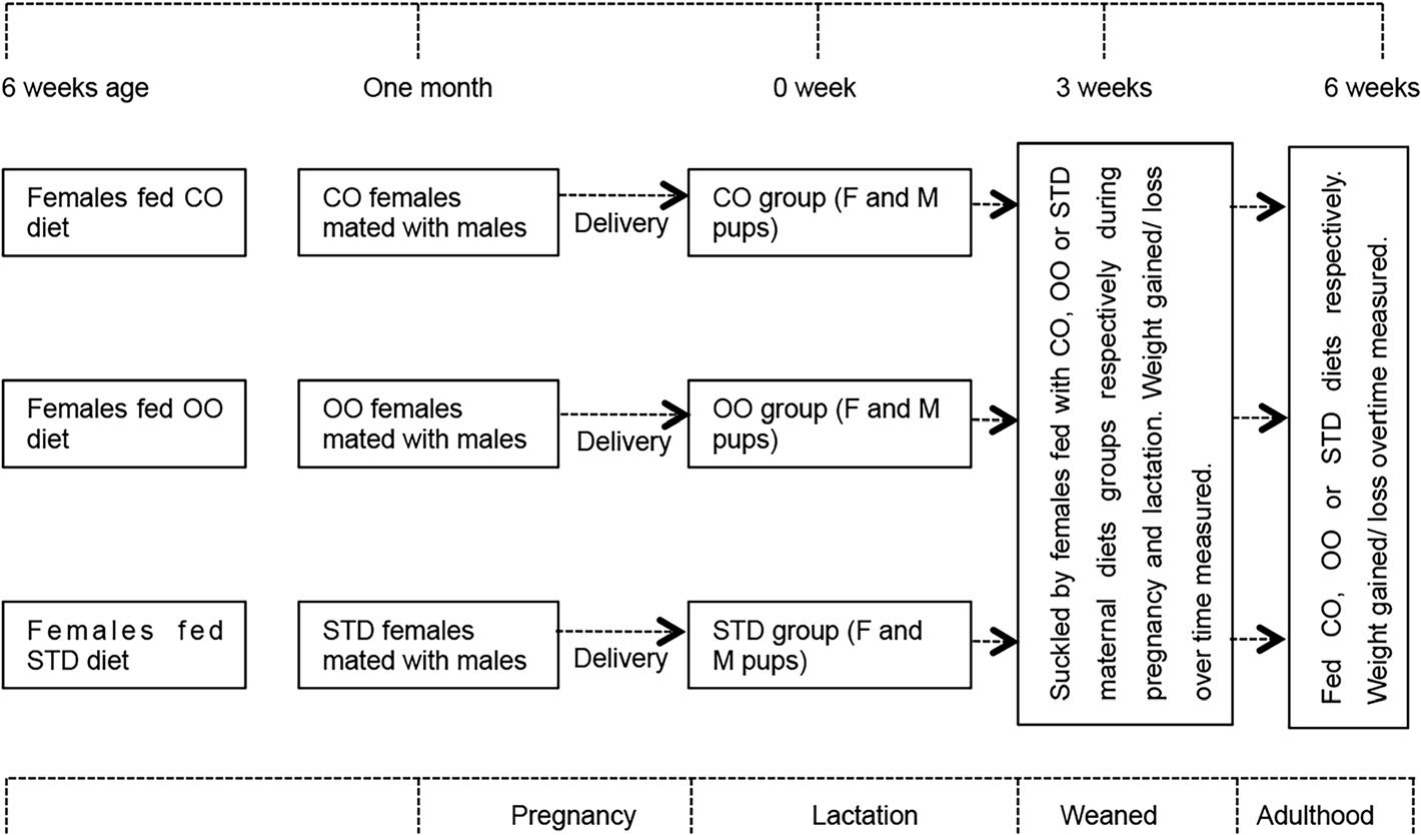
The study design and flowchart of experimental groups. Comparison between dietary groups. All pups in various experimental groups were delivered concurrently. For a duration of 1 month, grouped females were fed with standard (STD), virgin olive oil (OO) diet or virgin coconut oil (CO) diet. Completions of the absorption of the oil by the pellets were indicated by the change in the colour of soaked pellets with oil. At 3 weeks of age, the “spiky fur” phenotype developed in pups fed with coconut oil. Both control treatment, STD and OO showed no obvious differences in phenotype under the same growth condition. At the mature age of 6 weeks; the weight of CO pups is ominously reduced

Specified pathogen free outbred CD1 mice were used and maintained in an Animal Biosafety Level 2 (ABSL2) facility. Three groups (2 cages per group) of CD1 female mice were placed into different cages for their respective diet regimes at 6 weeks of age, mice at 6 weeks of age are mature and ready to be mated [26]. CD1 female mice were selected from the same litter with similar weight. Six cages were prepared whereby each cage contained three females. Two cages were given the virgin coconut oil diet which was designated as the experimental group. Control groups were two cages which were fed the standard diet and virgin olive oil diet, respectively. A flowchart of the procedure is as shown in Fig. 1.

### Diet preparation and feeding

Feeding commenced 1 month before planned mating between CD1 males and treatment groups’ females. Commercial cold-pressed virgin coconut oil imported from the Philippines (Brand: Country Farm) and virgin olive oil imported from the Italy (Brand: Bertolli) were used. The oil bottles were kept in the dark and stored in a refrigerator to avoid oxidation. The mouse pellets were soaked using a 10% w/w of the oil formula as previously described [27–29]. The oil-soaked pellets in our study contained 10% (w/w) fat, which was considered a workable compromise between the normal rat diet containing 5% (w/w) fat and the human diet with up to 35% of energy derived from fat [30, 31]. Information with the detailed composition and total energy for each diet is listed in Table 1. The diets were changed once every 3 days to minimise oxidation and to maintain the nutritional quality of the diet fed by the mice throughout the experiment. The food intake was measured throughout the study.

**Table 1:** Composition and total energy of each diet

^a^Composition	Extra virgin coconut oil (0.6 ml per serving)	Virgin olive oil (0.6 ml per serving)	Mouse pellets Barastoc Brand (Standard diet) (6 g per serving)
^c^Total energy (oil)	5.04 kcal 21.1 kJ	4.8 kcal 20.1 kJ	8.7 kcal 36.4 kJ
Crude protein	N/A	0 g	20.0%
Crude fat	0.6 g	0.6 g	5.0%
^b^Fatty acids Caprylic acid C8:0 Capric acid C10:0 Lauric acid C12:0 Palmitic acid C16:0 Elaidic acid C18:In9t Erucic acid C22:1n9 Docosadienoic acid C22:2 Docosahexaenoic acid C22:6n3 Linoleic acid C18:2n6c Alpla linoleic acid C18:3n3	7.2% 5.9% 43.5% 8.5% 7.8% 0.1% 1.0% 0.0% 6.3% 0.5%	0.1% 0.0% 0.2% 25.1% 0.0% 0.1% 0.1% 0.0% 9.2% 0.8%	0.1% 0.0% 0.5% 14.8% 20.0% 4.0% 9.7% 2.2% 21.7% 2.3%
Cholesterol	0 mg	0 mg	N/A
Crude fiber/ Carbohydrate	0 g	0 g	5.0%
Sugars	0 g	N/A	N/A
Salt	0 mg	0 mg	0.5%
Copper	N/A	N/A	7.5%
Selenium	N/A	N/A	0.1%
Calcium	N/A	N/A	0.8%
Total energy (pellets + 10% w/w oils)	12.87 kcal 53.8 kJ	12.63 kcal 52.8 kJ	8.7 kcal 36.4 kJ

Note: Energy provided based on 6 g consumption rate per day per mouse, ^a^composition of each diet based on manufacturer label, ^b^Fatty acids composition based on GCFID analysis, ^c^Thermochemical / food kilocalories to kilojoules: 1 kcal = 4.184 kJ, *N/A* data not available

### Mice mating

Timed-mating for all the groups was performed and the presence of the copulation plug the morning after the mating was taken as evidence of coitus.

### Measurements of weight gained/ loss overtime

The respective weight of each pup in the three study groups were measured daily using an electronic balance in order to measure the weight of the pups.

### Analysis of fatty acid in the diets

Pellets soaked in oils were left overnight to ensure complete absorption. The profile of the fatty acids of the standard pellets, virgin coconut oil and virgin olive oil immersed pellets respectively were analysed by gas chromatography flame ionization (GCFID) at the UnipeQ Food Research and Safety Unit, Universiti Kebangsaan Malaysia, Malaysia.

### Statistical analysis

The percentages of total fatty acids compositions were presented as the mean ± SEM. Graphs were generated and the level of weight was compared between groups using One-Way ANOVA followed by Bonferroni’s post-hoc test (Sigma plot version 12.0®). This test was used to compare between experimental groups and corresponding control groups, by analyzing continuous variables and to detect the differences of the mean values of weight gained/ loss over time.

## Results

### Virgin coconut oil causes low body weight during development

Pups of the three groups were delivered at 21 days after copulation. 39 pups were delivered from standard diet group, 27 pups were delivered from virgin olive oil group and 33 pups were delivered from virgin coconut oil group (Table 2). There is no significant difference in the litter size of mice fed the different diets. This indicates that the diets do not affect the fertility of the female mice.

**Table 2:** Average litter size (mean ± SE) for each treatment

Treatment	Litter 1	Litter 2	Litter 3	Mean ± SE
STD	15	10	14	13 ± 1.53
OO	11	8	8	9 ± 1.00
CO	12	9	12	11 ± 1.00

Note: *STD* standard diet, *OO* olive oil diet, *CO* coconut oil diet

**Fig. 2:**
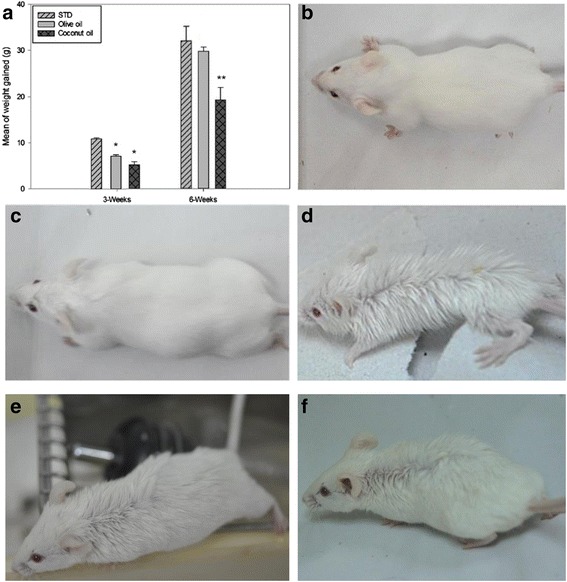
Comparison and phenotype of weight changes of dietary groups. **a**: We compared the weight between pups of the three groups at 3 and 6 weeks of age. Both OO- and CO- fed pups showed delay in growth and development compared to the STD fed pups at 3 weeks of age. Pups of control groups were significantly (*P* < 0.05) (One-way ANOVA) higher in weight over those fed with virgin coconut oil. Although weight of pups fed with virgin olive oil was significantly (*P* < 0.05) lower than those fed with STD at 6 weeks of age, pups fed with virgin olive oil had developed normally at late weaning stage of growth, due to insignificant (*P* > 0.05) differences in (One way ANOVA) weight gained corresponding to STD. Mann-Whitney Rank Sum Test shows that pups fed with virgin coconut oil were significantly (*P* < 0.05) delayed in growth to the virgin olive oil control and STD at 6 weeks of age. Single asterisk (*) represent significant differences between groups at 3-weeks of age. Double asterisk (**) represent significant differences between groups at 6-weeks of age. Values represent means, error bars are standard deviation. **b**: A typical CD1 mouse on normal diet. **c**: A typical CD1 mouse on virgin olive oil diet. **d**: A typical CD1 mouse on virgin coconut oil diet which exhibits the “spiky fur” coat phenotype and evidently skinnier than other littermates (*n* = 3 out of a total of 3 litters, *N* = 33). **e & f**: A typical mouse on virgin coconut oil diet which exhibits the “spiky fur” coat phenotype on its dorsal aspect

**Fig. 3:**
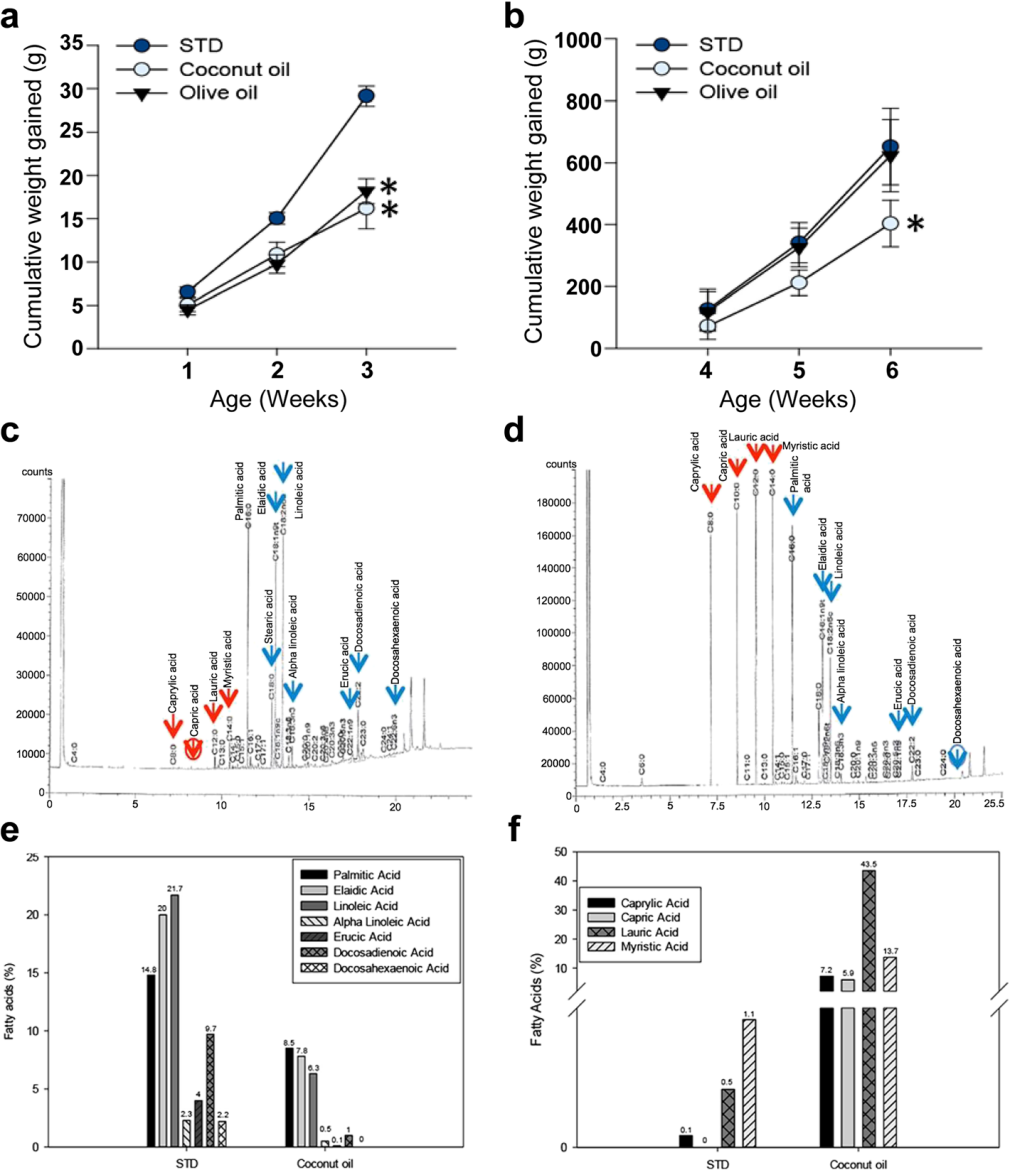
Fatty acid profile of the diets analysed by GCFID and alteration in the fatty acid profile of standard diet (Brand: Barastoc) when treated with virgin coconut oil as detected by GCFID. Fatty acids were transformed into fatty acid methyl ester via trans-esterification method. **a & b**: Growth curve showing increase in weight over time of which coconut oil diet shows consistent low body weight over time in comparison to standard diet. **a**: Measurement taken beginning at the onset of the dietary intervention until 3 weeks of age. **b**: Increase in weight until 6 weeks of age and showing data through the end of the experiment. *shows significant decrease in body weight in comparison to standard diet. **c**: The chromatogram shows the results of standard diet (STD) fatty acid profile; omega-6 linoleic acid, elaidic acid and palmitic acid appeared to be the highest in the profile of STD as indicated by *blue arrows*. **d**: The chromatogram shows the results of virgin coconut oil diet (CO) fatty acid profile; caprylic acid, capric acid, lauric acid and myristic acid were pronounced in its increase as indicated by *red arrows*. Elaidic acid, linoleic acid, alpha linoleic acid, erucic acid, docosadienoic acid and docosahexaenoic acid were visibly reduced, indicated by *blue arrows*. Docosahexaenoic acid was reduced from 2.2% in STD to 0% in virgin coconut oil diet, indicated by *blue circled arrow*. Capric acid increased from 0% in STD to 5.9% in virgin coconut oil diet, indicated by *red circled arrow*. **e**: Histogram showing representation of fatty acids from **c. f**: Histogram showing representation of change in fatty acids after virgin coconut oil treatment. Vast increase in the percentage of caprylic acid, capric acid, lauric acid and myristic acid, as a result, MCFAs of the total fatty acid profile of STD diet was raised. All data are based on laboratory analysis

Figure 2a shows the weight gained/ loss over time of the developing mice for the first 3 and 6 weeks of age before weaning/during lactation and maturity. Pups borne by females were maintained on their respective diets. No aversion to the oil-soaked pellets was seen as the volume of food consumed was similar, irrespective of diets (Table 3). There is no significant difference in mean of consumption rate (g per day) among diets (*P* > 0.05) The weight of pups fed with virgin coconut oil showed significantly low body weight when compared to corresponding control groups at 3 weeks of age and 6 weeks of age (*P* < 0.05) (Figs. 2a, 3a & b). Interestingly, pups of females fed with virgin olive oil also exhibited significantly low body weights compared to the standard diet group at 3 weeks of age (*P* < 0.05) (Figs. 2a & 3a). However, weight of pups fed with olive oil showed normal weight during maturity (6 weeks) in comparison to the standard diet mice (Figs. 2a & 3b); a comparison between the two diets proved insignificant (*P* > 0.05) (One-way ANOVA).

**Table 3:** Measurement of food intake (Mean ± SE) for each treatment

Treatment	Consumption rate (g per day)
STD	4.73 ± 0.16
CO	4.78 ± 0.10
OO	5.13 ± 0.13

Note: *STD* standard diet, *OO* olive oil diet, *CO* coconut oil diet

### Virgin coconut oil causes spiky fur phenotype

We observed that all of the 33 delivered pups treated with coconut oil developed irregularly arranged, odd-looking fur which we named as “spiky fur” (Fig. 2d, e & f). A typical phenotypic representation of the coconut oil diet mice are as shown in Fig. 2e & f. The mice were in general, healthy despite the unexplained dishevelled furs which were mostly spiky on the dorsal aspect of the mice (Fig. 2e & f). Among the 33 pups, three had pointedly spiky fur and was obviously skinnier than the rest. A typical pup is as shown in Fig. 2d. The three were somewhat lethargic and moved less robustly than its littermates although this was not measured. This phenotype did not develop in any the other pups fed with either virgin olive oil or standard diet under the same condition (Fig. 2b & c).

### Virgin coconut oil increases medium chain fatty acid of the diet

Fatty acid profile of standard and coconut oil diets were analyzed by GCFID as shown in Fig. 3c & d. Percentage of medium chain fatty acids (MCFAs) were highly elevated after standard pellets were soaked with coconut oil because of the increase of caprylic acid from 0.1 to 7.2%, capric acid from 0 to 5.9% and lauric acid from 0.5 to 43.5% (Fig. 3d, red arrows & f). Caprylic acid, capric acid and lauric acid are categorized under MCFAs [32]. Moreover, myristic acid was also altered to a high percentage from 1.1 to 13.7% (Fig. 3d & f). On the other hand, certain fatty acids were altered to a lower percentage such as palmitic acid from 14.8 to 8.5%, elaidic acid from 20 to 7.8%, erucic acid from 4 to 0.1%, docosadienoic acid from 9.7 to 1% and docosahexaenoic acid (DHA) from 2.2 to 0% (Fig. 3d, blue arrows & f). Interestingly, essential fatty acids (EFAs) such as linoleic acid and alpha linoleic acid were also reduced from 21.7 to 6.3% and 2.3% to 0.5%, respectively (Fig. 3d, blue arrows & f).

## Discussion

Coconut oil is known as a natural health product [33, 34]. No published study as yet has indicated the safety of coconut oil in pregnancy and infancy. Coconut oil is known to be a natural source of Medium Chain Triglycerides (MCTs) [35]. During digestion, MCTs will be converted to medium chain fatty acids (MCFAs) which cause weight loss and high energy expenditure [36]. In brief, fat is the primary source of energy [37], MCFAs metabolises quickly and lack the ability to be deposited as adipose tissue. This is because MCFAs are transported directly in the portal venous system and therefore bypasses peripheral tissues such as adipose tissue, therefore decreasing fatty deposition stores and lead to high energy expenditure [35]. Despite the fact that MCTs are known to be associated with low body weight in infants, it is still being used extensively as a source of energy for infant formulas and total parenteral nutrition [32, 35, 38]. The highly induced MCFAs in the diet could be the primary cause of the diminished weight during lactation and adulthood of the virgin coconut oil group, as in previous studies, animals fed with MCFAs had less weight gained associated with decreased fat deposition [36]. Caprylic acid, capric acid and lauric acid are fatty acids belonging to MCFAs [32, 39]. In our study, we noticed that the level of MCFAs is highly increased by the elevation in the concentration of caprylic acid, capric acid and lauric acid in the virgin coconut oil diet (Fig. 3d & f). Although caprylic acid does not have a direct effect on weight gain/loss; it does however influence the mechanism of acylation of ghrelin which modulates appetites and feelings of satiety [40]. Furthermore, both capric acid and lauric acid promotes fatty acid oxidation which directly increases satiety and therefore have a direct effect on weight gain/loss [41]. Besides that, the reduction in the weight gain observed may be influenced by the genotype of the animal. The dietary administration of MCTs may influence mRNA expression of genes capable of modifying the lipogenic ability of the animal; therefore altering the phenotype of the animal [42]. MCTs are agents of body fat reduction which works via the peroxisome proliferator activated receptor-γ by down-regulating adipogenic genes. It is interesting to note that the effect of MCFAs have an abiding effect on body weight reduction although the mechanism often involves an increase in fasting cholesterol as well as triglyceride levels. There is a certain degree of dispute in this whereby MCFAs have been noted to reduce fasting lipid levels provided MCFAs are given in moderate amounts in the form of moderate fat supply [43]. We are the first to examine the changes in fatty acids profile of diets altered with the addition of common cooking oils, and thus the first to study the association of low body weight in weaned pups and matured mice of the virgin coconut oil diet.

Coconut oil had caused a significant decrease in weight of pups compared to standard and virgin olive oil fed pups (Fig. 2). Our study examined virgin coconut oil which is rich in MCTs (Fig. 3), in particular, established virgin coconut oil feeding on infant development, body weight and other phenotypes during lactation and until maturity. Our study provides extra evidence for the use of MCTs oil (such as coconut oil) as part of a weight-loss program. In recent studies, it was suggested that MCTs diet could be useful for controlling body weight gained fat in healthy adult subjects [44] but we suggest treating the use of coconut oil during pregnancy and infancy with caution. The MCTs had no toxicological properties even with long-term feeding diets [44]. Nevertheless, MCTs adversely alters lipid profile by increasing total plasma cholesterol, higher low density lipoprotein cholesterol and higher plasma total triglycerol [45].

Other than MCTs, the potential mechanism for the decrease in growth and development of pups fed with coconut oil diet may be due to the deficiency in docosahexaenoic acid (DHA) that is essential for infants’ growth [46, 47]. MCFAs have also been found to secrete peptide YY (PYY) which causes postprandial satiety signalling in mammals although this is potentially secreted in response to long chain triglycerides as well [48]. Since, our study focussed on the fatty acid profile of oil and their relation to lower body weight, more detailed studies on the metabolic enzymes and relevant hormone levels changes in mice due to oil consumption needs to be done in the future. It is known that vegetables prepared with raw oils help increase the absorption of nutrients such as carotenoid [49, 50]. MCTs oil has also been used to treat impaired absorption of long-chain fats in patients [51]. MCTs consumption causes slight elevation in triglyceride level in children with malabsorption syndromes [52].

Furthermore, this is the first report of a spiky fur phenotype in mice although previous literature have shown that in the absence of essential amino acids and certain proteins, the spiky fur phenotype have been observed in rats [53]. This information tallies with our GCFID data on the coconut oil itself which shows a much lowered amount of essential fatty acids in comparison to olive oil and the non-oil treated control. We postulate therefore that in the absence or reduced amounts of both essential amino acids and essential fatty acids, the mice suffer in its coat quality.

We have shown evidence that the likelihood of virgin coconut oil for dietary consumption during and after pregnancy may cause harmful effects to the developing pup until maturity of the mice. On the basis of our findings, we stress that coconut oil as herbal medicine for pregnant women, infants and growing children need to be studied more intensively.

## Conclusion

Experimentally, we have shown in this study that virgin coconut oil alters the essential fatty acids profile and lipid profile in standard mouse pellet; thereby inducing high levels of MCFAs/ MCTs. We observe that this change then in turn causes low body weight and changes quality of fur coat of mice which we have coined as the spiky fur phenotype. Our findings warrant further investigation and monitoring of coconut oil usage in women of child-bearing years. Therefore, we report that the use of coconut oil during pregnancy for prolonged periods should be assessed with caution until sufficient data and information becomes available.

## Abbreviations

DHA: Docosahexaenoic acid
EFAs: Essential fatty acids
GCFID: Gas chromatography flame ionization detector
MCFAs: Medium chain fatty acids
MCTs: Medium chain triglycerides
PYY: Peptide YY

## References

[1] Ab Rahman A, Ahmad Z, Naing L, Sulaiman SA, Hamid AM, Daud WN (2007). The use of herbal medicines during pregnancy and perinatal mortality in Tumpat District, Kelantan, Malaysia. Southeast Asian J Trop Med Public Health.

[2] Dugoua J (2010). Herbal medicines and pregnancy. J Popul Ther Clin Pharmacol.

[3] Ernst E (2002). Herbal medicinal products during pregnancy: are they safe?. BJOG-Int J Obstet Gy.

[4] Tiran D (2003). The use of herbs by pregnant and childbearing women: a risk-benefit assessment. Complement Ther Nurs Midwifery.

[5] Pinn G, Pallett L (2002). Herbal medicine in pregnancy. Complement Ther Nurs Midwifery.

[6] Trigt AM, Waardenburg CM, Haaijer-Ruskamp FM, Jong-van den Berg LTW (1994). Questions about drugs: how do pregnant women solve them?. Pharm World Sci.

[7] Chan L, Chiu P, Lau T (2003). An in vitro study of ginsenoside Rb1 induced teratogenicity using a whole rat embryo culture model. Hum Reprod.

[8] Ernst E, White A (2000). The BBC survey of complementary medicine use in the UK. Complement Ther Med.

[9] Nordeng H, Havnen GC (2004). Use of herbal drugs in pregnancy: a survey among 400 Norwegian women. Pharmacoepidemiol Drug Saf.

[10] Noordalilati MN, Sulaiman SA, Sembulingam K, Afifi SAB (2004). Evaluation of the teratogenicity study of standardized extract of *Andrographis paniculata* in rats. Proceedings of the seminar on medicinal and aromatic plants: Current trends and perspectives.

[11] Chin RKH (1991). Ginseng and common pregnancy disorders. Asia Oceania J Obstet Gynaecol.

[12] Chan K (2003). Some aspects of toxic contaminants in herbal medicines. Chemosphere.

[13] Morris G, Mdlalose B (1991). The use of isihlambezo in the upper Tugela region. S Afr Fam Prac.

[14] Chuang CH, Lai JN, Wang JD, Chang PJ, Chen PC (2006). Use of Coptidis Rhizoma and foetal growth: a follow up study of 9895 pregnancies. Pharmacoepidemiol Drug Saf.

[15] Veale D, Havlik I, Katsoulis L, Kaido T, Arangies N, Olive DW (1998). The pharmacological assessment of herbal oxytocics used in South African traditional medicine. Biomed Environ.

[16] Islam M, Sulaiman SA, Kapitonova MY, Jamallullail SMS (2007). Effects of an indigenous contraceptive herbal formulation on gonadotrophs of the pituitary gland of the rat. Malays J Med Sci.

[17] Mabina M, Pitsoe S, Moodley J (1997). The effect of traditional herbal medicines on pregnancy outcome. The King Edward VIII Hospital experience. S Afr Med J.

[18] St-Onge MP, Lamarche B, Mauger JF, Jones PJH (2003). Consumption of a functional oil rich in phytosterols and medium-chain triglyceride oil improves plasma lipid profiles in men. J Nutr.

[19] Sbragia L, Nassr ACC, Gonçalves FLL, Schmidt AF, Zuliani CC, Garcia PV (2014). VEGF receptor expression decreases during lung development in congenital diaphragmatic hernia induced by nitrofen. Braz J Med Biol Res.

[20] Beauchamp GK, Keast RSJ, Morel D, Lin J, Pika J, Han Q (2005). Phytochemistry: ibuprofen-like activity in extra-virgin olive oil. Nature.

[21] Rahman AA, Sulaiman SA, Ahmad Z, Daud WNW, Hamid AM (2008). Prevalence and pattern of use of herbal medicines during pregnancy in Tumpat district, Kelantan. Malays J Med Sci.

[22] Fife B (2005). Coconut cures: preventing and treating common health problems with coconut.

[23] Fife B (2005). Procedures, formulas and recipes. Coconut cures: Preventing and treating common health problems with coconut.

[24] Liau KM, Lee YY, Chen CK, Rasool AHG (2011). An open-label pilot study to assess the efficacy and safety of virgin coconut oil in reducing visceral adiposity. ISRN Pharmacol.

[25] Kim Sooi L, Lean KS (2013). Herbal medicines: Malaysian women’s knowledge and practice. Evid Based Complement Alternat Med.

[26] Heifetz Y, Wolfner MF (2004). Mating, seminal fluid components, and sperm cause changes in vesicle release in the *Drosophila* female reproductive tract. Proc Natl Acad Sci U S A.

[27] Iritani N, Fukuda E, Kitamura Y (1980). Effect of corn oil feeding on lipid peroxidation in rats. J Nutr.

[28] Al-Amoudi NS, Abu Araki HA (2013). Evaluation of vegetable and fish oils diets for the amelioration of diabetes side effects. J Diabetes Metab Disord.

[29] Palit P, Furman BL, Gray AI (1999). Novel weight-reducing activity of Galega officinalis in mice. J Pharm Pharmacol.

[30] Hasani-Ranjbar S, Nayebi N, Larijani B, Abdollahi M (2009). A systematic review of the efficacy and safety of herbal medicines used in the treatment of obesity. World J Gastroenterol.

[31] Siemelink M, Verhoef A, Dormans JA, Span PN, Piersma AH (2002). Dietary fatty acid composition during pregnancy and lactation in the rat programs growth and glucose metabolism in the offspring. Diabetologia.

[32] Traul K, Driedger A, Ingle D, Nakhasi D (2000). Review of the toxicologic properties of medium-chain triglycerides. Food Chem Toxicol.

[33] Fife B (2006). Coconut oil and health. Coconut revival: new possibilities for the ‘tree of life’. Proceedings of the International Coconut Forum in Cairns, Australia.

[34] Fife B (2002). The healing miracles of coconut oil.

[35] Clegg ME (2010). Medium-chain triglycerides are advantageous in promoting weight loss although not beneficial to exercise performance. Int J Food Sci Nutr.

[36] Noguchi O, Takeuchi H, Kubota F, Tsuji H, Aoyama T (2002). Larger diet-induced thermogenesis and less body fat accumulation in rats fed medium-chain triacylglycerols than in those fed long-chain triacylglycerols. J Nutr Sci Vitaminol (Tokyo).

[37] Coggan AR, Raguso CA, Gastaldelli A, Sidossis LS, Yeckel CW (2000). Fat metabolism during high-intensity exercise in endurance-trained and untrained men. Metabolism.

[38] St-Onge MP, Bosarge A (2008). Weight-loss diet that includes consumption of medium chain triacylglycerol oil leads to a greater rate of weight and fat mass loss than does olive oil. Am J Clin Nutr.

[39] Shahidi F, Smith J, Charter E (2010). Functional and nutraceutical lipids. Functional Food Product Development.

[40] Lemarié F, Beauchamp E, Legrand P, Rioux V (2016). Revisiting the metabolism and physiological functions of caprylic acid (C8:0) with special focus on ghrelin octanoylation. Biochimie.

[41] Lemarié F, Beauchamp E, Dayot S, Duby C, Legrand P, Rioux V (2015). Dietary caprylic acid (C8:0) does not increase plasma acylated ghrelin but decreases plasma unacylated ghrelin in the rat. Plos One.

[42] Ferreira L, Lisenko K, Barros B, Zangeronimo M, Pereira L, Sousa R (2014). Influence of medium-chain triglycerides on consumption and weight gain in rats: a systematic review. J Anim Physiol Anim Nutr (Berl).

[43] Marten B, Pfeuffer M, Schrezenmeir J (2006). Medium-chain triglycerides. Int Dairy J.

[44] Nagao K, Yanagita T (2010). Medium-chain fatty acids: functional lipids for the prevention and treatment of the metabolic syndrome. Pharmacol Res.

[45] Tholstrup T, Ehnholm C, Jauhiainen M, Petersen M, Høy CE, Lund P (2004). Effects of medium-chain fatty acids and oleic acid on blood lipids, lipoproteins, glucose, insulin, and lipid transfer protein activities. Am J Clin Nutr.

[46] Al MD, van Houwelingen AC, Hornstra G (2000). Long-chain polyunsaturated fatty acids, pregnancy, and pregnancy outcome. Am J Clin Nutr.

[47] Lee HS, Barraza-Villarreal A, Biessy C, Duarte-Salles T, Sly PD, Ramakrishnan U (2014). Dietary supplementation with polyunsaturated fatty acid during pregnancy modulates DNA methylation at IGF2/H19 imprinted genes and growth of infants. Physiol Genomics.

[48] Maas MIM, Hopman WPM, Katan MB, Jansen JBMJ (1998). Release of peptide YY and inhibition of gastric acid secretion by long-chain and medium-chain triglycerides but not by sucrose polyester in men. Eur J Clin Investig.

[49] Conlon LE, King RD, Moran NE, Erdman JW (2012). Coconut oil enhances tomato carotenoid tissue accumulation compared to safflower oil in the Mongolian gerbil (*Meriones unguiculatus*). J Agric Food Chem.

[50] Goltz SR, Sapper TN, Failla ML, Campbell WW, Ferruzzi MG (2013). Carotenoid bioavailability from raw vegetables and a moderate amount of oil in human subjects is greatest when the majority of daily vegetables are consumed at one meal. Nutr Res.

[51] Bach AC, Babayan VK (1982). Medium-chain triglycerides: an update. Am J Clin Nutr.

[52] Tamir I, Gould S, Fosbrooke AS, Lloyd JK (1969). Serum and adipose tissue lipids in children receiving medium-chain triglyceride diets. Arch Dis Child.

[53] Plahar WA, Okezie O, Annan NT (2003). Nutritional quality and storage stability of extruded weaning foods based on peanut, maize and soybean. Plant Foods Hum Nutr.

